# CRISPR targeting of SNPs associated with age-related macular degeneration in ARPE-19 cells: a potential model for manipulating the complement system

**DOI:** 10.1038/s41434-025-00522-z

**Published:** 2025-03-18

**Authors:** Ahmed Salman, Won Kyung Song, Tina Storm, Michelle E. McClements, Robert E. MacLaren

**Affiliations:** 1 Nuffield Department of Clinical Neurosciences, University of Oxford, Oxford, UK; 2 Oxford Eye Hospital, Oxford University Hospitals NHS Foundation Trust, Oxford, UK; 3 Present Address: Gangnum Yonsei Eye Clinic, Seoul, Republic of South Korea

**Keywords:** Visual system, Genetic vectors, Gene regulation

## Abstract

Age-related Macular degeneration (AMD) is a major cause of vision loss and is linked to several predisposing single nucleotide polymorphisms (SNPs). CRISPR-mediated genome editing offers the potential to target negatively associated SNPs in an allele-specific manner, necessitating the need for a relevant cell model. The ARPE-19 cell line, with its stable monolayer growth and retinal pigment epithelium (RPE) characteristics, serves as an ideal model for AMD studies. Chronic inflammation and complement system dysregulation are implicated in AMD pathogenesis. Most genetic variations associated with AMD are in complement genes, suggesting their regulatory role. In this study, we conducted targeted PCRs to identify AMD-related SNPs in ARPE-19 cells and used CRISPR constructs to assess allele-specific activity. Guide RNA sequences were cloned into an EF-1-driven SpCas9 vector and packaged into lentivirus. Targeting efficiencies were evaluated with TIDE analysis, and allele-specificity was measured with NGS analysis 30 days post-transduction. Our results showed varying targeting efficiencies depending on guide RNA efficacy. For example, TIDE analysis of CFH SNPs rs1061170 and rs1410996 revealed efficiencies of 35.5% and 33.8%, respectively. CFB SNP rs4541862 showed efficiencies from 3% to 36.7%, and rs641153 ranged from 3.4% to 23.8%. Additionally, allele-specific targeting of AMD-related SNPs rs1061170, rs1410996, rs4541862, and rs641153 ranged from 48% to 52% in heterozygous differentiated ARPE-19 cells. These findings demonstrate the potential to manipulate the complement system in an AMD model by targeting disease-associated SNPs in an allele-specific manner, offering a promising therapeutic approach.

## Introduction

Age-related Macular Degeneration (AMD) is the leading cause of irreversible central vision loss in the developed world [1, 2]. It accounts for 8.7% of blindness worldwide with 255 million affected individuals expected in the next 25 years. The accumulation of drusen between the retinal pigment epithelium (RPE) and Bruch’s membrane in the retina is a key phenotypic feature of AMD. The pathogenesis of AMD progresses through early, intermediate and advanced stages, the latter being divided into two categories: neovascular (wet), characterised by blood vessel growth, and non-neovascular (dry). Increasing evidence suggests that AMD involves chronic inflammatory processes, with the complement system playing a significant role in its pathogenesis [3]. Complement proteins, such as Complement Factor H (CFH), have been shown to be involved in the formation and biogenesis of drusen. Several studies have also identified genetic variants of CFH that are associated with an increased risk of AMD [4–7], suggesting its regulatory role in the disease.

Although various cell models are used in acute macular degeneration research such as RPE-J, MI0-M1, and hTERT [8–10], these cell lines tend to lose RPE characteristics after multiple passages in culture. ARPE-19, a human immortalised cell line that arose spontaneously from a primary RPE culture from a 19-year-old male [11], offers a distinct advantage. The epithelia morphology of these cells combined with their rapid proliferation rate, distinguishes them from other primary RPE cultures and makes them a useful model for RPE studies. Unlike most immortalised cell lines derived from primary cultures, which tend to lose their specialised properties, ARPE19 cells retain their RPE morphology even after multiple passages. Differentiated ARPE19 cells grow in a stable monolayer, maintain expression of known RPE and differentiation markers, and form tight junctions, a well-characterised property of the RPE [12].

The complement system is an important part of the innate immune system, involving over forty proteins. It is responsible for a wide range of immune activities, such as eliminating microbes, clearing immune complexes, and modulating the adaptive immune response (reviewed in [13]). The complement system is activated by three major pathways: (i) The classical pathway, (ii) The lectin pathway and (iii) The alternative pathway, which converge at the level of complement component 3 (C3) convertase, leading to the cleavage of C3, the central component of the complement cascade, into C3a and C3b. C3 and C5 can be further cleaved to amplify the cascade and initiate the terminal pathway, leading to the formation of the Membrane Attack Complex (MAC) [14].

The classical pathway, which uses complement component 1 (C1), is triggered by antigen-antibody immune complexes. In its inactive form, C1 circulates in the blood as a complex of six Cq molecules and two serine protease attachments (C1r and C1s) [15]. When C1 binds to a cognate antigen, it undergoes a conformational change, activating C1r and C1s. activated C1s then cleaves complement components 4 and 2 (C4 and C2), leading to the formation of the classical pathway C3 convertase C4b/C2a complex, which cleaves C3 into C3a and C3b (see Supplementary Fig. 1). In addition to being activated by antigen-antibody immune complexes, Cq1 can also be activated by apoptotic and necrotic cells, as well as C-reactive proteins [16]. The lectin pathway mirrors the classical pathway, as its activation also results in the formation of the C3 convertase C4b/C2a complex. However, instead of relying on antigen-antibody complexes, the lectin pathway is activated by members of the collectin family of plasma proteins, such as mannose-binding lectins and ficolins [17]. These proteins bind to sugar molecules on the surface of invading pathogens, which then leads to the cleavage of C4 and C2 and the formation of the C3 convertase C4b/C2a complex, similar to the classical pathway. The alternative pathway is in a “*tick over”* state, meaning it is continuously active at low levels [15]. Unlike the classical and lectin pathways, the alternative pathway does not require exogenous stimuli, such as antigen-antibody complexes or mannose residues. Activation of the alternative pathway induces a conformational change in C3 and the binding of factor B (CFB), initiating the alternative pathway C3 convertase via an amplification loop (see Supplementary Fig. 1).

The complement system plays a key role in host defence against pathogens but must be tightly regulated to prevent inflammation and tissue damage [18]. The involvement of the complement system in AMD pathogenesis has long been documented, although the exact mechanisms remain unclear (reviewed in [19]). It is speculated that an inadequately regulated complement cascade resulting in unwanted immune responses, may contribute to AMD pathogenesis. This theory is supported by the detection of immune complexes in drusen. Photoreceptor degeneration in the macula is a direct secondary consequence of disruption of the RPE layer, which can become a target for activated complement proteins [20, 21].

AMD has a strong genetic component, with several genetic variants, or single nucleotide polymorphisms (SNPs), linked to an increased risk of the disease. Many of these variants are found in genes that encode complement factor proteins, such as complement factors H, I, and B, as well as complement components C2, C3 and C9. These variants span the allelic spectrum of the disease, ranging from common variants with a low impact on disease pathogenesis to rare variants with nearly complete penetrance.

In 2005, a genome-wide association study (GWAS) identified a highly significant genetic variant in CFH (Tyr402His) [4], a finding corroborated by other studies [5, 22, 23]. Over the next decade, additional studies discovered variants in CFI, CFB C2, C3 and C9 [24–29]. Moreover, a common haplotype carrying a deletion in the complement factor H-related genes *CFHR1* and *CFHR3* was found to be protective against AMD [30]. In 2016, Fritsche and colleagues conducted a GWAS to identify variants independently associated with AMD. Of the 52 variants they identified (45 common and 7 rare), more than a third (19 variants) were within the complement system [24], further highlighting the association between SNPs in the complement system with AMD pathogenesis. Despite the strong genetic link to AMD, with heritability estimated at 40-70% in dizygotic twins, predicting the cause remains difficult due to the multifactorial nature of the disease. Environmental factors, such as smoking, the most common environmental factor [31], can also play a significant role in AMD pathogenesis.

The recent discovery of the CRISPR-Cas9 mechanisms in the bacterial immune system, and its subsequent adaptation into a powerful gene editing tool, has revolutionized molecular biology and sparked excitement about the potential for novel therapeutic approaches to treat human conditions [32]. There are two classes of CRISPR systems: Class 1 and Class 2 [33]. The Cas9 protein, which acts as a single effector protein, is derived from the Class 2 system. Class 1 and Class 2 CRISPR systems require CRISPR RNA (crRNA) for target specificity. Additionally, some Class 2 variants, such as type II systems, which include the Cas 9 protein, require an additional trans-activating RNA (tracrRNA) that forms a scaffold structure [34]. The crRNA and the tracrRNA are often fused into a single small guide RNA (sgRNA), which, along with Cas9, forms the Cas9:sgRNA complex. This complex scans the DNA for the appropriate protospacer adjacent motif (PAM), a short motif adjacent to the target site. Upon recognizing the PAM, Cas9 unwinds the DNA, allowing the sgRNA to bind to its complementary exposed DNA, the protospacer. This pairing activates the catalytic domains of the Cas9 protein, inducing double-stranded breaks (DSBs) [35].

In eukaryotic cells, Cas9-induced DSBs are repaired by one of two mechanisms: non-homologous end joining (NHEJ) or homology-directed repair (HDR) [36, 37]. NHEJ is the typical repair mechanism for Cas-induced DSBs, it is efficient and prevalent but often results in random insertions and deletions (indels) that disrupt the target DNA. However, NHEJ is also error-prone. In contrast, HDR provides a precise restoration of the target DNA, leading to fewer off-target events (alterations of unintended DNA regions). Despite its precision, HDR is less efficient than NHEJ due to the requirement for a correct DNA template for repair. The CRISPR-Cas system includes a variety of components that differ widely in their mechanisms of action and offer therapeutic potential through direct genome interaction and/or editing. Variations in haplotypes of different complement genes are associated with dysregulated levels of complement activation complements [38]. Disease variants typically result in increased levels of complement activation components, while protective variants are associated with decreased levels. This variation in complement activation markers presents a potential target for CRISPR manipulation. For example, using CRISPR-cas9 to introduce indels that knock down a disease-causing variant can potentially result in a normal or protective haplotype [39].

Influencing the expression profile of pathogenic or protective SNPs by targeting specific alleles may offer a potential mechanism for controlling the complement cascade in AMD. One of the main challenges in targeting AMD risk variants in the complement system with CRISPR is achieving allele specificity of the disease variants.

Due to the human-specific nature of AMD, achieving an appropriate in vitro model to assess treatment options has proven challenging. However, the ARPE-19 cell line has been shown to secrete complement factors [40], making it a potential in vitro model for studying AMD. In this study, we demonstrate the successful targeting of various AMD-related SNPs in the complement system using a CRISPR approach. We investigated the challenges and limitations in achieving allele specificity when targeting SNPs with this method, highlighting the potential of using this model for future diagnostic and therapeutic applications in manipulating the complement system in AMD.

## Materials and methods

### ARPE-19 cell culturing and differentiation

ARPE-19 cells (ATCC, CRL 2302 Lot #: 63478793) were cultured in GlutaMAX Dulbecco’s Modified Eagle Medium (DMEM; Gibco) F-12 mixture (Ham), supplemented with 10% (v/v) Foetal Bovine Serum (FBS), 100 U/mL penicillin, and 0.1 mg/mL Streptomycin (Gibco), at 37 C in a humidified atmosphere of 5% CO^2^. For differentiation, cells were seeded at a density of 90000 cells/cm^2^ in Primaria non-pyrogenic polystyrene coated 96-well plates (353872; Scientific Laboratory Supplies). Twenty-four hours after seeding, cells were differentiated following the method described by Samuel et al. [12], in high glucose DMEM (Gibco) supplemented with 1% (v/v) FBS, 200 mM L-Glutamine and 100 U/mL penicillin, and 0.1 mg/mL Streptomycin, at 37 C in a humidified atmosphere of 5% CO^2^ for up to three months. The media was changed twice weekly with freshly prepared media.

### Immunocytochemistry

ARPE-19 cells were seeded in Corning Primaria 96-well plates and cultured or differentiated as described above. Cells were washed twice with phosphate-buffered saline (PBS; 10010015, Gibco) and fixed with 4% methanol-free formaldehyde (28906; Thermo Fisher Scientific) for 15 min. Cells were then permeabilised with 0.1% Triton x-100 diluted in PBS and supplemented with 1% bovine serum albumin (BSA) for 10 min at room temperature. After permeabilisation, cells were incubated with rabbit anti-ZO-1 antibody (1:200) (617300; Thermo Fisher Scientific) for 1 h at room temperature and visualised with a secondary goat anti-rabbit Alexa Fluor™ 488 (1:1000) (A11008; Thermo Fisher Scientific). Cell nuclei were counterstained with Hoechst 33342 (H1399; Thermo Fisher Scientific) for 5 min at room temperature. Bright-field images were captured using an inverted epifluorescence microscope (DMIL; Leica). Immunofluorescence staining was captured with EVOS FL Auto 2.0 imaging platform (Thermo Fisher Scientific). Images were processed with ImageJ.

### Karyotyping

Metaphase spreads were harvested following synchronisation with 0.1 mg/ml nocodazole (Merck; M1404). The cells were swollen in buffered hypotonic solution (Genial Helix; GGS-JL006) and fixed in Carnoy’s fixative. For chromosome counting, slides were mounted in VECTASHIELD Antifade Mounting Medium with DAPI (2-B Scientific; H-1200-10). Multiplex fluorescence in situ hybridisation (M-FISH) was performed using the 24XCyte probe-set (Metasystem Probes; D-0425-060DI), following the manufacturer’s instructions. ChromaTide™ Alexa Fluor™ 594-5-dUTP or ChromaTide™ Alexa Fluor™ 488-5-dUTP (ThermoFisherer Scientific; C11400 and C11397, respectively) were incorporated into a set of representative human probes. The probes were hybridised using a standard protocol [41]. All cytogenetics preparations were analysed using the Leica Cytovision software, on an Olympus BX-51 epifluorescence microscope, equipped with a JAI CVM4+ progressive-scan 24 fps B&W fluorescence CCD camera.

### Guide RNA design and cloning

The plenti-EFS-SpCas9-EGFP-U6-gRNA plasmid was a gift from Dr Lucy McDermott, University of Oxford (Addgene; Plasmid 82416). Single-guide RNAs (gRNAs) were designed using Geneious software (USA). All guides were 20 bp in length, with the selected SNPs positioned 5–7 bp of the PAM (Supplementary Table 3). gRNAs were subcloned into the BsmBl restriction site. In this study, the gRNAs targeted SNPs rs1410996, rs1061170, rs380390, rs641153, rs541862 and rs147259257. For gRNA cloning, 100 mM oligos were phosphorylated for 20 min, then annealed with 1.5 °C dropping temperature series from 95 °C down to 25 °C using a thermocycler (SimpliAmp; Thermofisher). The annealed/phosphorylated guides were ligated to the linearised pLenti-FSH-SpCas9-EGFP-U6-gRNA vector after digestion with BsmBl enzyme (New England BioLabs). Ligated vectors were transformed into NEB Stable cells (New England BioLabs). The ligated vectors were then transformed into NEB stable cells (New England BioLabs), and colonies were picked for culture expansion in Luria Broth. For Lentivirus production, endotoxin-free plasmids were prepared using the NeocleoBond Endotoxin-free Maxiprep kit (Machery-Nagel).

### Lentivirus production and transduction

Lentivirus was produced in HEK293T cells (ACCT, CRL 11268) using a 3^rd^ generation lentivirus production system, incorporating psPAX2 and pMDG.2 packaging and plasmids (gift from Dr Lucy McDermott, University of Oxford) in combination with the pLenti-EFS-SpCas9-EGFP-U6-gRNA plasmid. Cells were seeded on 0.001 Poly-L-Lysine coated T75 flasks in 15 mL of high glucose DMEM (Gibco) supplemented with 10% (v/v) FBS, 200 mM L-Glutamine, 100 U/mL penicillin and 0.1 mg/mL Streptomycin, at 37 C in a humidified atmosphere of 5% CO^2^. Twenty-four hours after seeding, cells were co-transfected with psPAX2, pMDG.2 and the pLenti-EFS-SpCas9-EGFP-U6-gRNA in a 4:2:1 ratio (respectively) using Lipofectamine 3000 transfection reagent (Thermo Fisher). After twelve hours, the transfection reagent was removed and replaced with high glucose DMEM (Gibco) supplemented with 10% (v/v) FBS and 200 mM L-Glutamine, without antibiotics. The supernatant containing the lentiviral particles was collected twice after twenty-four and forty-eight hours and filtered through a 0.45 μM membrane filter (Merck Millipore). Lentivirus particles were purified by ultracentrifugation using Amicon Ultra-15 100 K filter units (Merck Millipore). One month differentiated ARPE-19 cells were plated in a 96 well plate and transduced with 100 uL diluted lentivirus stock (1:5 dilution of lentivirus stock containing pLenti-EFS-SpCas-U6-gRNAs in high glucose DMEM (Gibco) supplemented with 1% (v/v) FBS, 200 mM L-Glutamine and 100 U/mL penicillin and 0.1 mg/mL Streptomycin). Cells were left transduced for one month with media changes twice weekly.

### PCR amplification and TIDE analysis

Genomic DNA was extracted from the transduced ARPE-19 cells using the QIAamp DNA mini kit (Qiagen). Polymerase chain reaction (PCR) was performed to amplify the relevant region using KOD HotStart Mastermix (Merck Millipore) with primers designed to amplify approximately 600–700 bp fragments containing the target SNP (Supplementary Table 3). The PCR products were purified using the QIAquick PCR purification kit (Qiagen) and analysed on an agarose gel to confirm a clean amplification before being subjected to Sanger sequencing (Eurofins Genomics). DNA editing efficiency was measured by uploading chromatograms to the Tracking by Indels Decomposition (TIDE) online software [42] to calculate indel percentages. Decomposition windows and left boundaries were optimized based on the distance of the cut sites to achieve the highest alignment for each target. Indel sizes were kept at 10 indels, and the significance cut-off was at *p* < 0.001 for all analyses.

### Data analysis

TIDE analysis was employed to determine the spectrum and frequency of indels in transduced ARPE-19 cells. The TIDE online database (https://tide.nki.nl) was used to upload Sanger sequencing files (.ab1) and gRNA sequences, with chromatograms from non-transduced cells serving as controls. The alignment window for the left boundary was set at 100 bp, and the right boundary was set to the automatic break site of −10 bp. The decomposition window was initially set between 110–580 bp; if the gRNA binding site fell outside this range, the window was adjusted accordingly. The Indel size range was set at 10, and the *P*-value threshold for significance was set at 0.001. For next-generation sequencing quantification (Eurofins Genomics), nucleotide quantification windows were measured by uploading chromatograms into *CRISPResso2* software [43]. Chromatograms from non-transduced cells were used as controls. The quantification window was optimised using the *Cas9* custom editing tool, with Paired-end reads and a minimum homology alignment of 60%, setting the centre of the amplification window at −10 and the quantification window size to 1 bp.

## Results

### Targeting efficiencies for different AMD-related SNPs

**Fig. 1:**
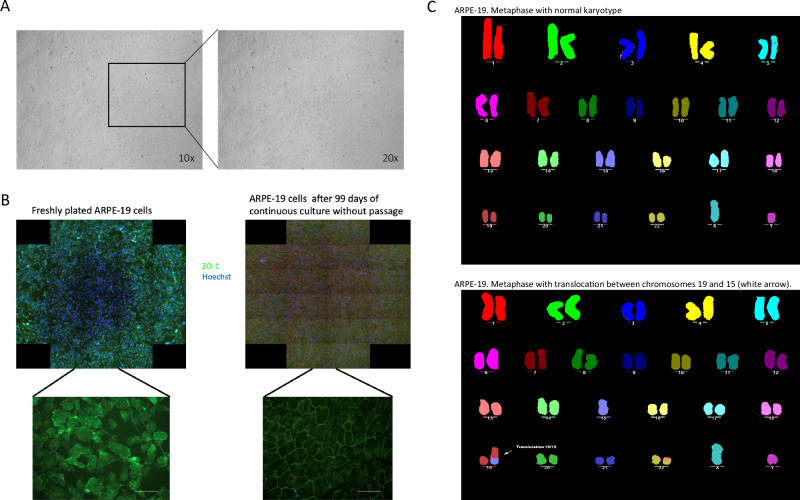
Cultured ARPE-19 cells exhibit normal morphology and ploidy. **A** Phase contrast images of one month differentiated ARPE-19 cells in culture display typical retinal pigment epithelium (RPE) morphology, characterised by a uniform monolayer and distinct tight-junctions. Images taken at 10X and 20X magnifications. **B** comparison of freshly plated ARPE-19 cells 99-day differentiated, non-passaged cells, cells were stained for Zo-1 (green) to highlight tight-junctions, and Hoechst 3334 (blue) as a nuclear counterstain. **C** Multicolour fluorescence in situ hybridisation (MFISH) analysis of sub-confluent ARPE19 cells using fluorescent probes against human chromosomes. This analysis revealed a normal modal number of *n* = 46, with a translocation between chromosomes 15 and 19, and part of chromosome 13 translocated to chromosome 22.

Differentiated ARPE-19 cells in 96-well plates should display a cobblestone epithelial morphology, and a tight, uniform monolayer with no visible holes [11, 12]. Our differentiated ARPE-19 cells exhibited the expected characteristics at one month, as shown in Fig. 1A, demonstrating a uniform monolayer with distinct tight junctions, evidenced by ZO-1 staining (Fig. 1B). Cell transformation is often associated with aneuploidy and other chromosomal abnormalities, which are common in spontaneously immortalised cells. Therefore, we examined the karyotype of ARPE-19 cells (passage 9) (Fig. 1C). The analysis confirmed that the ARPE-19 cells used in this study were diploid with normal chromosomal structure, except for a small translocation in chromosome 19 (arrow in Fig. 1C), which was present in approximately 25% of the cell population. This particular translocation has been reported in the literature previously [44]. Screening for chromosomal abnormalities is crucial because, like any immortalised cell line, ARPE-19 cells can exhibit mosaicism for structural chromosomal aberration that may vary between laboratories. Ploidy is especially important for downstream applications, such as CRISPR/Cas9 targeting, as genomic editing is more challenging in hyperdiploid genomes [45] due to the need to edit all functional copies of a gene to achieve complete editing or knockout.

Next, we assessed whether the ARPE-19 cells carried AMD-related SNPs in selected complement factor genes. We performed PCR genotyping targeting CFH, CFB, C2, C3 and C5, and identified 21 different SNPs in these genes (Supplementary Table 1), with varying statuses of homozygosity and heterozygosity. This variability provided an ideal opportunity for manipulating the complement cascade using CRISPR/Cas targeting. To examine this, we cloned gRNAs targeting different SNPs (Supplementary Fig. 2A) into the pLenti-U6-gRNA-EFS-SpCas9-EGFP vector and packaged them into Lentivirus particles. The CRISPR-Cas9 Type II system derived from *Streptococcus pyogenes* (SpCas9) is among the most well-characterised and widely used CRISPR-Cas systems [46, 47]. The human U6 RNA promoter is commonly employed for highly ubiquitous RNA polymerase-mediated expression of gRNA [48, 49].

**Fig. 2:**
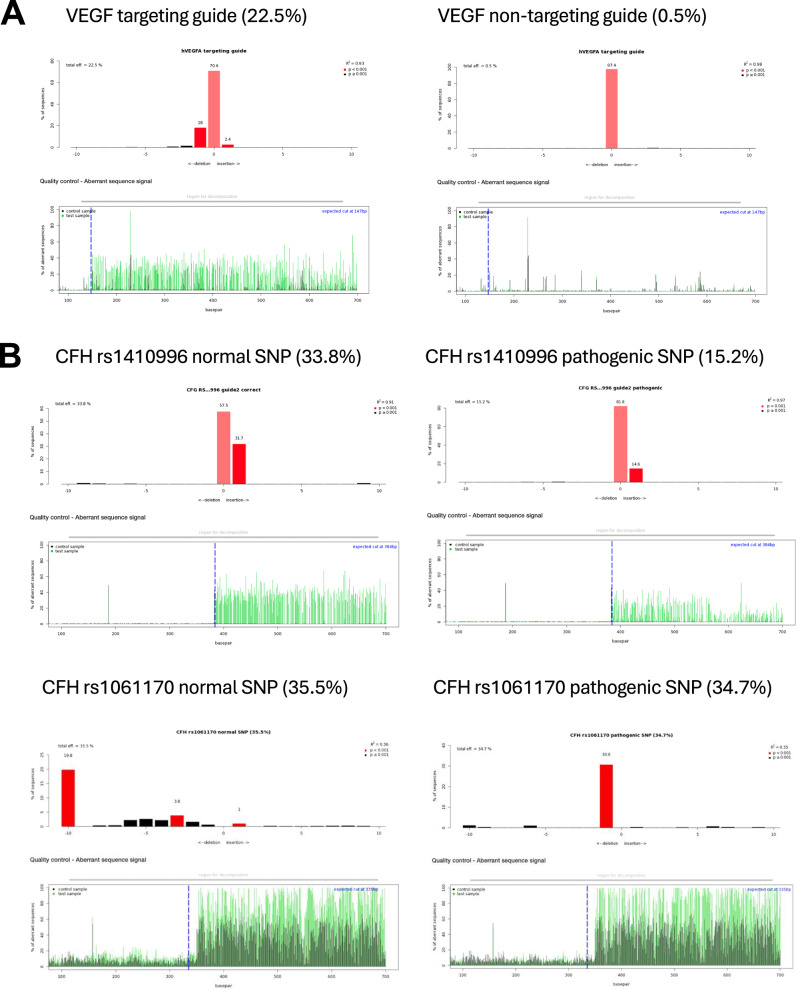

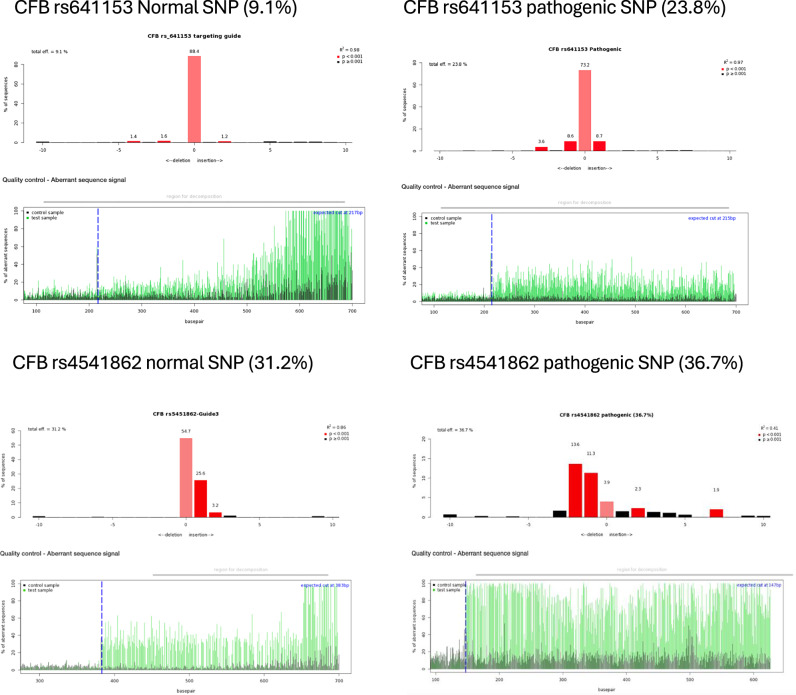
Targeting efficiency of CRISPR constructs in transduced ARPE-19 cells. **A** Representative diagrams from Tracking of Indels by Decomposition (TIDE) analysis output, showing indels in sequencing chromatograms of one month transduced ARPE-19 cells with CRISPR vectors with guide RNA targeting the vascular endothelial growth factor (VEGF) gene as a control (*N* = 3). **B** TIDE analysis for CRISPR constructs with guide RNAs targeting AMD-related SNPs in various complement genes (*N* = 3). Red columns indicate the percentages and locations of insertions/deletions (indels) with a *P* value < 0.001, while black columns represent indels with a *P* value > 0.001. The blue dotted lines mark the Cas9 enzyme cut site, and the green lines indicating disruptions in the DNA sequence after the cut.

To investigate the editing efficiency of gRNAs, ARPE-19 cells were transduced with lentivirus encapsulating the pLenti-U6-gRNA-EFS-SpCas9-EGFP vector (Supplementary Fig. 2B). The editing rates reached up to 36%, as judged by TIDE analysis (Table 1). A published VEGF gRNA was used as a positive control (Fig. 2A). Transfections of gRNA into differentiated ARPE-19 cells yielded lower editing rates (Supplementary Table 2). For the AMD-related SNPs, each gRNA was tested in two forms: one targeting the normal (healthy) allele and the other targeting the AMD-associated (pathogenic) allele. The efficiency of editing varied depending on the position and proximity of the PAM relative to the target SNP (Fig. 2B and Table 1).

**Table 1:** TIDE efficiencies of normal and pathogenic AMD-related SNPs of the complement cascade in 1-month transduced ARPE-19 cells.

SNP	Editing rate
CFB rs641153 guide 2-Wild type	9.10%
CFB rs641153-guide 2-Pathogenic	23.80%
CFB rs641153-guide 3-Wild type	11.90%
CFB rs641153-guide 3-Pathogenic	13.50%
CFB rs641153-guide 1-rv -Wild type	17.30%
CFB rs641153-guide 1-rv-Pathogenic	3.40%
CFB rs541862-guide 1-Wild type	3%
CFB rs541862-guide 1-Pathogenic	3.40%
CFB rs541862-guide 2-Wild type	3.10%
CFB rs541862-guide 2-Pathogenic	3.20%
CFB rs541862-guide 3-Wild type	31.20%
CFB rs541862-guide 3-Pathogenic	36.70%
CFH rs1410996-guide 2-Wild type	33.8%
CFH rs1410996-guide 2-Pathogenic	23.50%
CFH rs1061170-guide 1-Wild type	35.50%
CFH rs1061170-guide 1 Pathogenic	34.70%

### Evidence of allele specific CRISPR editing of AMD risk SNPs in ARPE-19 cells

**Fig. 3:**
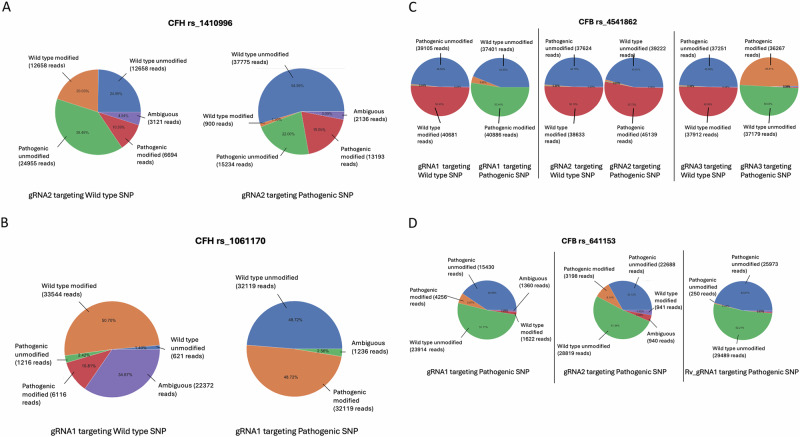
Next-generation sequencing data analysis of CRIPSR-targeted ARPE-19 cells. Pie charts representing next-generation sequencing data from one month transduced ARPE-19 cells (*n* = 3) targeted with gRNAs against either the wild type or pathogenic alleles of CFH rs1410996 (**A**), CFH rs1061170 (**B**), CFB rs541862 (**C**), and CFB rs641153 (**D**). The charts highlight the percentages of modified versus unmodified alleles in each case.

TIDE analysis provided an indication of the editing efficiency of each gRNA, but achieving a precise targeting approach for AMD-risk SNPs requires a deeper understanding of the allele specificity, which is crucial for future therapeutic applications. Therefore, next-generation sequencing (NGS) was performed at the target sites to assess specificity. NGS data from ARPE-19 cells transduced with gRNAs targeting different SNP variants were analysed using a pipeline to determine allele specificity. For instance, a 50% editing rate for a target SNP in heterozygous ARPE-19 cells (i.e., containing both wild type and pathogenic variants) would indicate that only one allele was edited, assuming the gRNA edited only the target allele. When targeting SNPs, near-complete allele specificity was observed, with editing occurring predominantly at the target allele and minimal editing at the other. For example, when targeting the pathogenic form of the CFH risk variant rs1410996, 19.05% editing occurred in the pathogenic allele compared to only 1.3% in the wild type allele (Fig. 3A). Conversely, targeting the wild type allele, editing occurred primarily in the wild type allele (20.03%), however, editing in the pathogenic allele was also observed at 10.59%. This preferential editing of the target allele was also observed when targeting the wild type allele of the CFH risk variant rs1061170, the most common AMD-related SNP [4, 5, 22, 23], NGS analysis showed allele-specific editing of 50.70% for the wild type allele (Fig. 3B). Similarly, when targeting the pathogenic form, 48.72% of editing occurred in the pathogenic allele.

A similar trend was observed with the CFB risk variant rs541862, where editing predominantly occurred at the target allele, with minimal edits at the non-target allele, regardless of whether the wild type or pathogenic allele was targeted. For example, when targeting the wild type allele with one gRNA (gRNA1), 50.45% editing was observed in the wild type allele, compared to just 0.53% in the pathogenic allele (Fig. 3C).

This trend remained consistent across different gRNAs. For instance, gRNA2 exhibited near-complete allele specificity when targeting the wild type allele, with 50.10% editing observed in the wild type allele compared to just 0.80% in the pathogenic allele. Similarly, when targeting the pathogenic allele, 52.72% editing was achieved with only 0.80% editing in the wild type allele (Fig. 3C). The same pattern was observed with gRNA3 (Fig. 3C), where 49.95% editing was achieved when targeting the wild type allele, compared to just 0.34% in the pathogenic allele. Similarly, 50.03% editing was achieved when targeting the pathogenic allele, compared to 0.38% in the wild type counterpart.

The pattern of allele specificity observed with SNPs was most pronounced near the PAM site. However, when the SNP was located further away from the PAM site, as seen with RV gRNA1 targeting the CFB protective variant rs641153, the degree of allele specificity varied (Fig. 3D). With gRNAs 1 and 2 achieving editing rates of 5.67% and 9.14% (respectively) in the target allele.

**Fig. 4:**
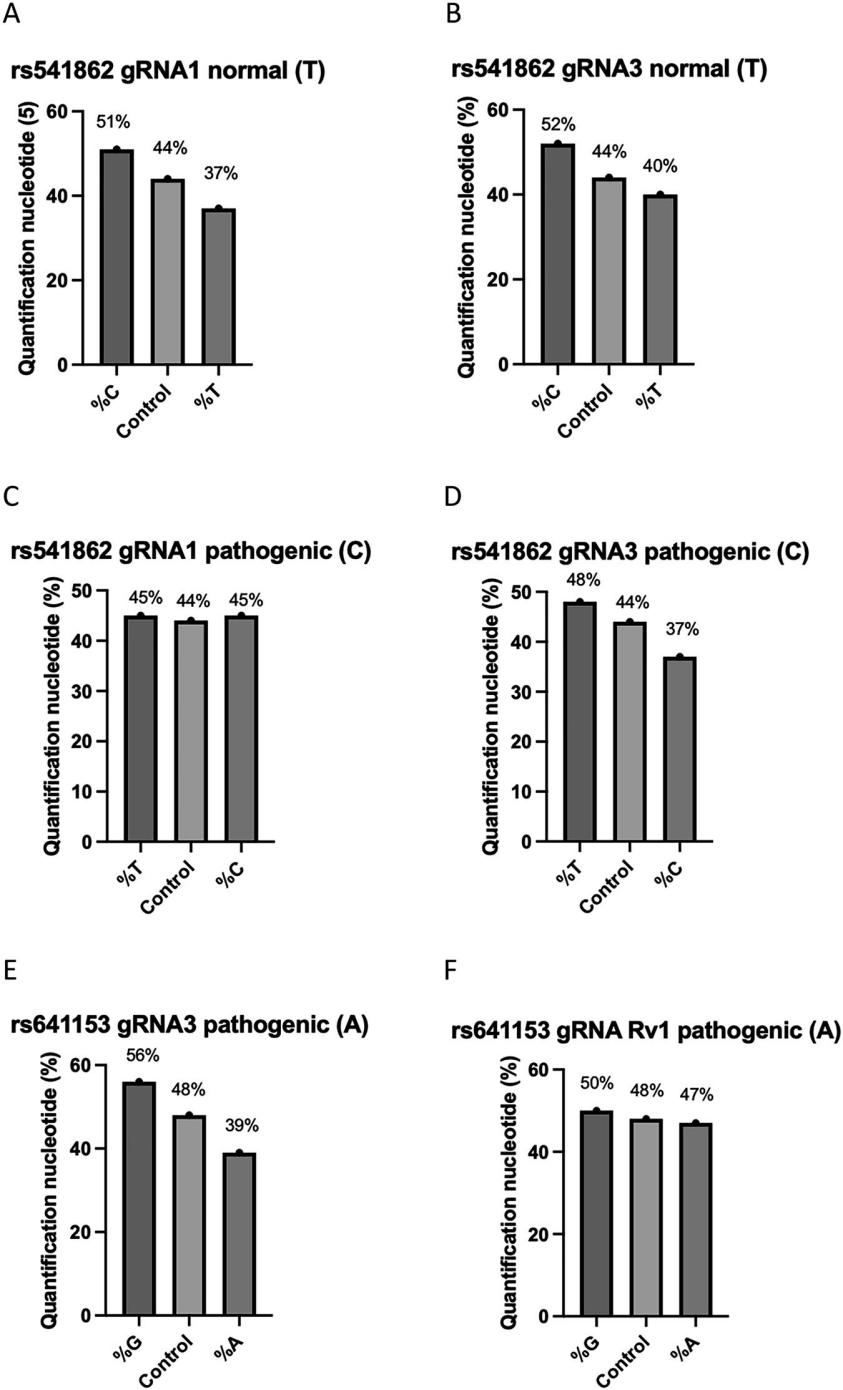
Percentages of allele specificity of AMD-related SNPs. Quantification plots showing percentage changes in normal and pathogenic variants compared to the control following CRIPSR editing. **A** rs541862 (normal, T) targeted with gRNA1. **B** rs541862 (normal, T) targeted with gRNA3. **C** rs541862 (pathogenic, C) targeted with gRNA1. **D** rs541862 (pathogenic, C) targeted with gRNA3. **E** rs641153 (pathogenic, A) targeted with gRNA3. **F** rs641153 (pathogenic, A) targeted with gRNA Rv1 (*n* = 3).

Targeting normal SNPs led to a shift towards a higher percentage of pathogenic SNPs while targeting pathogenic SNPs resulted in an increase in the percentage of normal SNPs (Fig. 4A–F). For example, targeting the normal (T) SNP in rs541862 reduced its percentage to 37% and 40% with gRNAs 1 and 3, respectively, compared to 44% in the control. Concurrently, the percentage of the pathogenic (C) allele changed to 51% and 52%, with the same gRNAs (Fig. 4A, B).

Conversely, targeting the pathogenic (C) allele led to a balanced contribution with approximately 45% for both pathogenic and normal alleles (C and T respectively) with gRNA1. However, with gRNA3, the pathogenic (C) allele decreased to 37%, while the normal (T) allele increased to 48%, compared to 44% in the control (Fig. 4C, D).

For rs641153, targeting the pathogenic (A) SNP decreased the percentage of the (A) allele to 39% and 47% with gRNA 1 and Rv1, respectively, compared to 48% in the control. Concurrently, the percentages of the normal (G) allele increased to 56% and 50% with the same gRNAs (Fig. 4E, F).

## Discussion

This study explores a novel CRISPR targeting approach to manipulate the complement cascade in AMD using the reliable ARPE19 cell line as an in vitro model. Our results demonstrate efficient DNA editing with CRISPR in ARPE-19 cells, with notable allele specificity in the editing of various AMD-related SNPs in the complement system. The key factor influencing allele specificity is the distance of the target SNP from the PAM site.

In recent years, allele-specific CRISPR genome editing has emerged as a promising therapeutic approach for treating genetic diseases with specific risk variants (reviewed in ref. [50]). Traditionally, allele-specific interventions have been achieved using short-interfering RNAs (siRNAs) or antisense oligonucleotides, but the CRISPR system offers several advantages. First, CRISPR allows for precise genome editing capable of distinguishing between pathogenic and wild type alleles. Second, it provides the flexibility to generate PAMs derived from genetic variants, enabling the targeting of any gene locus, rather than being limited to transcribed mRNA (for a more detailed review see ref. [50]).

The proximity of the targeted SNP from the PAM site appears to be the main factor influencing allele specificity. In this study, most of the gRNAs targeted SNPs located within 1 to 6 base pairs from the PAM site, except for one (rs641153 gRNA rv1). For those SNPs, near-complete allele specificity was achieved in transduced ARPE-19 cells. Analysis of NGS data from PCR amplicons targeting SNPs such as rs1410996 and rs1061170 (both in CFH), rs541862 and the protective rs641153 (both in CFB) showed strong preference for editing the targeted allele, with minimal editing of the non-target allele.

For example, SNPs positioned between one to seven base pairs from PAM site, such as gRNA2 of rs1410996 (five base pairs), gRNA1 of 1061170 (two base pairs), gRNA1 of rs541862 (one base pair) or gRNA1 and gRNA2 targeting rs641153 (six to seven base pairs), exhibited high allele specificity. However, when the SNP was further away from the PAM site, as with Rv-gRNA1 targeting rs641153 (19 base pairs), allele specificity was significantly reduced or absent.

Occasionally, the specificity of certain gRNAs was suboptimal. For instance, with gRNA2 targeting the wild type allele of rs1410996, some editing also occurred in the pathogenic allele, despite a preference for the wild type. Several factors may explain this, including (i) variation in gRNA efficiencies based on SNP-PAM distance, (ii) random indels with varying lengths introduced by Cas9, leading to misreads and reduced on-targeting editing, and (iii) differences in NGS sequencing quality, which is crucial for determining allele specificity.

The seed region near the PAM site, especially the PAM-proximal 10–12 bases (3’ end), is crucial for Cas9 cutting activity [51, 52]. However, the extent of mismatch tolerance varies across gRNAs, and a defined seed region may not apply uniformly across all gRNAs. While mismatches near the PAM region are generally less tolerable, the distal regions of the gRNA can tolerate mismatches without disrupting DNA cleavage [53]. In genome-wide studies, a clearly defined seed region, particularly the first five nucleotides near the PAM, was identified in one out of two and three out of four gRNAs tested [54, 55]. Studies on SpCas9 specificity show contrasting results regarding mismatch tolerance between the gRNA and the target DNA (gRNA:DNA). In general, mismatches at the PAM-distal (5’ terminal) end of the gRNA tend to be more tolerable, often not affecting DNA cleavage. While mismatches at the PAM-proximal (3’ terminal) region are less tolerable, typically disrupting DNA cleavage by SpCas9.

Jinek et al. demonstrated that up to six mismatches in the PAM-distal region could be tolerated without disrupting cleavage, while even a single mismatch in the PAM-proximal disrupted activity [35]. Similarly, Cong et al. found that up to nine bases at the PAM-distal region were tolerable to gRNA:DNA mismatches, but mismatches within the first 11 bases of the PAM-proximal end completely disrupt DNA cleavage [52]. Hsu et al. further described variable gRNA:DNA tolerance across all 20 bases of the gRNA [56], and Doench et al. found that the rG:dT (where T appears in the target DNA with G, instead of A, in the complementary position on the gRNA) is the most tolerable gRNA:DNA mismatch [57]. In this study, most of the gRNAs targeting SNPs were located within five to seven bases of the PAM site, gRNA:DNA mismatches in this region may have affected specificity, making allele specific targeting a challenge (reviewed in ref. [58]). The gRNA:DNA mismatch tolerance, combined with the presence or absence of highly specific subregions within the seed sequence, complicates achieving perfect allele specificity. One potential solution is the use of high-fidelity Cas nucleases with improved specificity, along with SNP-derived PAM sites to ensure edits occur only when the target SNP is present in the PAM.

The most significant percentage change was observed in rs641153 when targeting the pathogenic variant (A) with gRNA 1, which resulted in 8% increase in the normal (G) allele compared to the control. Since the ARPE-19 cells are heterozygous for this protective SNP, a roughly equal percentage of each allele is expected. The increase in the percentage of the normal (G) allele suggests that random indels led to a small percentage of the targeted pathogenic (A) allele, around 8%, being converted to the normal (G) allele, indicating that the CRISPR approach was successful. However, these changes in allele percentages are estimates, since factors such as variable lengths of random indels generated by Cas9 editing, and off-target events make it difficult to obtain accurate estimates of exact base changes. While sequencing read errors cannot be entirely ruled out, next-generation sequencing is typically highly reliable and accurate. Historically, DNA sequencing relied on Sanger sequencing methods [59], which was used to detect rare variants. However, next-generation sequencing has revolutionized the field ever the last decade, making DNA sequencing analysis faster and quantifiable, particularly for amplifying targeted SNPs regions via PCR, as used in this study. Despite the potential of CRISPR for direct DNA editing and SNP correction, this approach used has limitations, including low efficiency of targeting for SNPs located outside an appropriate seed region, and high rate of undesired indel mutations. CRISPR-Cas mediated base-editing systems (or ”base editing”) and the newer prime editing systems offer solutions to these challenges, holding considerable potential as therapeutic tools for repairing disease-causing SNPs.

To conclude, this study provides proof-of-principle evidence that CRISPR technology can be used to manipulate the complement cascade in an AMD model, laying the groundwork for future translational applications in treatment, and prevention of, AMD. While the physiological impact of editing AMD-related variants in the complement system remains unknown, our data demonstrate, at the molecular level, that these variants can be manipulated in an allele specific manner using a reliable AMD model. Genetic variations in the complement system play a significant role in AMD pathogenesis. However, functional studies are needed to further interpret these genetic findings and expand our understanding of the complement system’s role in AMD. By leveraging the precision and specificity of CRISPR-Cas mechanisms to target disease predisposing SNPs, this approach holds potential as an effective therapeutic tool to correct disease-causing variants and address the underlying mutations that contribute to AMD.

## Supplementary information

Supplementary materials

## Data Availability

The datasets generated during and/or analysed during the current study are available from the corresponding author on reasonable request. Targeted SNPs can also be found on European Variation Archive (EVA).
