# Supplementary material for: CRISPR targeting of SNPs associated with age-related macular degeneration in ARPE-19 cells: a potential model for manipulating the complement system
Source: Gene Ther. 2025 Mar 18;32(2):132–41. doi: 10.1038/s41434-025-00522-z (PMC11946884; doi:10.1038/s41434-025-00522-z)
Supplement: Supplementary file 1 — Supplementary materials [file 41434_2025_522_MOESM1_ESM.docx]

**CRISPR targeting of SNPs associated with Age-related Macular Degeneration in ARPE-19 cells: a potential model for manipulating the complement system**

Ahmed Salman^1^, Won Kyung Song^1,2**^, Tina Storm^1^, Michelle E. McClements^1^ and Robert E. MacLaren^1,2*^

^1^Nuffield Department of Clinical Neurosciences, University of Oxford, Oxford, UK.

^2^Oxford Eye Hospital, Oxford University Hospitals NHS Foundation Trust, Oxford, UK

^*^Corresponding author

** Now at the Gangnum Yonsei Eye Clinic, Seoul, Republic of South Korea

**Supplementary materials**


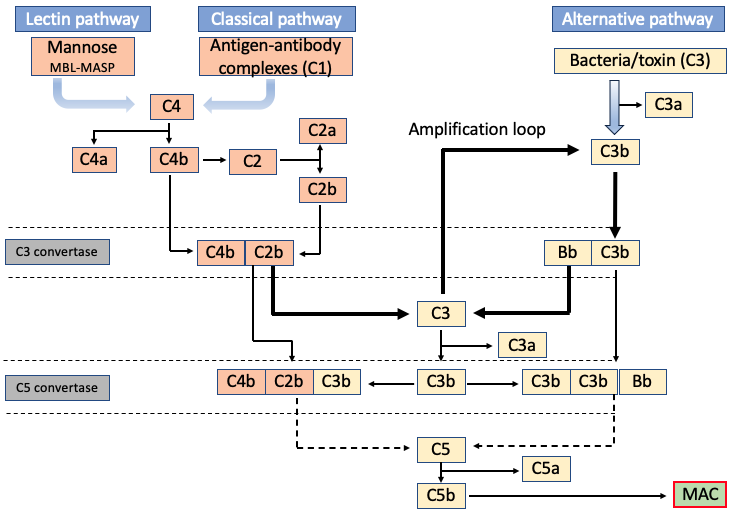


**Supplementary Figure 1**. **Schematic diagram of the complement cascade**. The complement cascade initiated by three primary pathways: the lectin pathway and the classical pathway (both shown in orange), and the alternative pathway (yellow). All pathways converge at the C3 and C5 convertases (grey). Activation of the classical pathways begins with the recruitment of C1 complex, which cleaves C4 and C2 into C4a, C4b and C2a, C2b, respectively. The lectin pathway is similar to the classical pathway but is triggered by mannose residues. Activation of the alternative pathway, from which most AMD-related variants are derived, starts with the hydrolysis of circulating C3 into C3b. A confirmational change in C3 allows for binding of CFB, initiating the formation of the alternative pathway C3 convertase. C3b also plays a role in the formation of C5 convertase, which is subsequently cleaved into C5a and C5b, leading to the formation of the membrane attack complex (MAC).

**A**


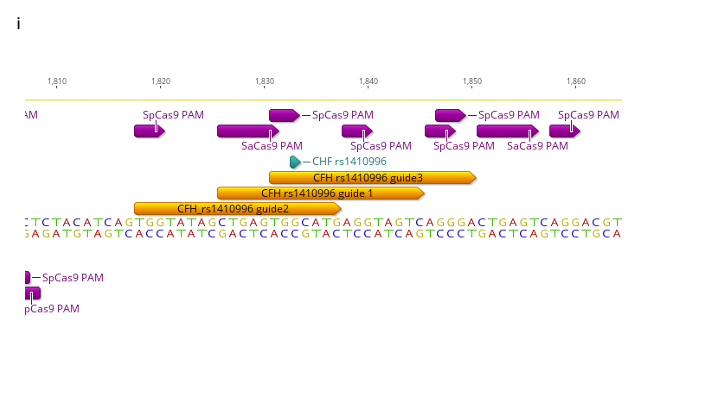

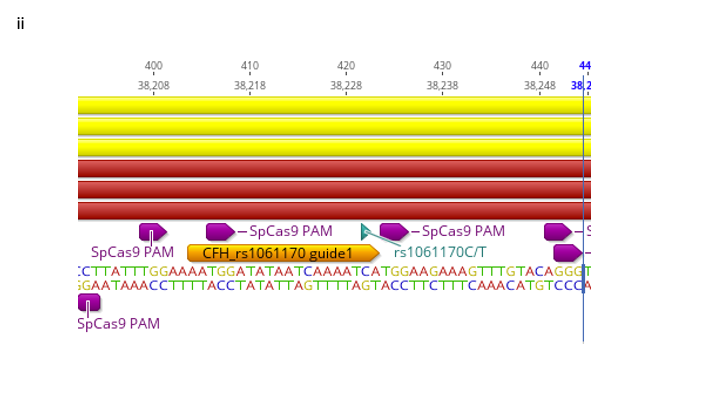


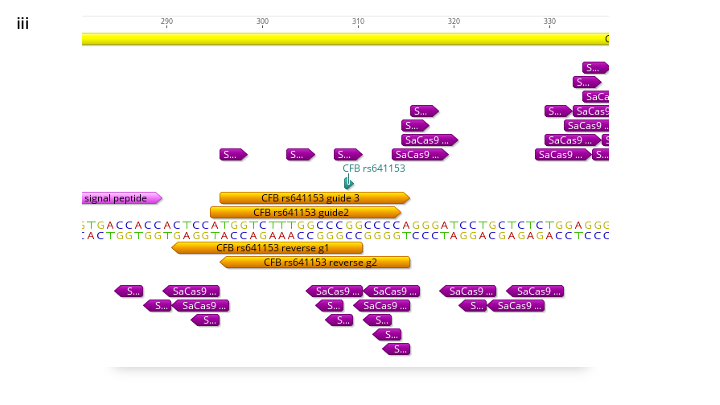

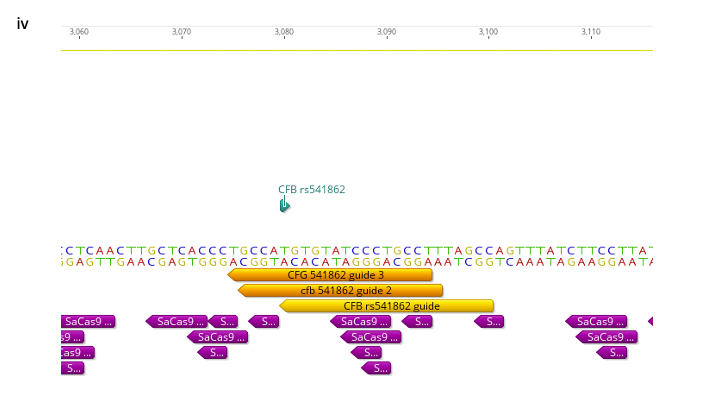


**B**

10x

**
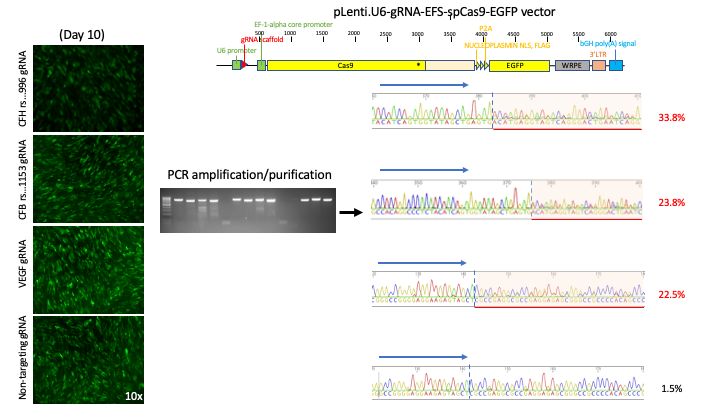
**

**Supplementary Figure 2. Workflow of CRISPR manipulation of AMD-related SNPs in ARPE-19 cells.** (**A**)Representative sequence diagrams showing the positions of selected AMD-related SNPs in complement genes: (i) rs1410996, (ii) rs1061170; (iii) rs641153, and (iv) rs4541862, along with the orientations and locations of the gRNAs used for lentivirus transductions in ARPE-19 cells. (**B**) The workflow schematic outlines the CRISPR targeting of AMD-related SNPs, showing one month differentiated ARPE-19 cells transduced with lentivirus encapsulating gRNAs targeting these SNPs. PCR amplification and Sanger sequencing chromatograms display sequence disturbances caused by CRISPR-induced random indels, visible in cells transduced CRISPR constructs with gRNAs targeting CFH rs1410996, CFB rs641153 and hVEGF, showing editing efficiencies of 31.2%, 17.3% and 22.5%, respectively. No sequence disturbance is observed in the non-targeting control, with only 1.5% editing efficiency.

**Supplementary Table 1. Major AMD-related SNPs in the complement cascade in ARPE-19 cells.**

| **AMD related SNPs** | **Seq result in ARPE19 DNA** | **consequence** | **pathogenic** | **Allele freq.** |
| --- | --- | --- | --- | --- |
| CFH Rs1061170 C>T | hetero C/ T | TYR402HIS | O | C 0.32133 |
|  |  | NP_000177.2:p.His402Tyr missense variant |  | T 0.67867 |
| CFH Rs10733086 A>C / A>T | hetero A/T | Intron variant NA | O | A=0.36313 |
|  |  |  |  | T=0.63687 |
| CFH Rs1831282 G/T(reverse A>C) | hetero A/C | Intron variant NA | O | A=0.36184 |
|  |  |  |  | C=0.63816 |
| CFH Rs35292876 C>T | homo C | NM_000186.3(CFH):c.2634C>T (p.His878=) | X | C=0.98949 |
|  |  | Coding seq variant, synonymous variant |  | T=0.01051 |
| CFH Rs1329424 A/C(r T>G) | hetero T/G | Intron variant NA | O | T=0.32267 |
|  |  |  |  | G=0.67733 |
| CFH Rs10737680 A>C | hetero A/C | Intron variant NA | O | A=0.56526 |
|  |  |  |  | C=0.43474 |
| CFH Rs121913059 C>T | homo C | R1210C; NP_000177.2:p.Arg1210Cys missense variant | X | C=0.99986 |
|  |  |  |  | T=0.00014 |
| CFH Rs380390 G>A / G>C / G>T | hetero G/C | Intron variant NA | O | G=0.3586 |
|  |  |  |  | T=0.0001, C=0.6413 |
| CFH Rs10801555 F1R2 A>G | hetero A/G | Intron variant NA | O | A=0.34119 |
|  |  |  |  | G=0.65881 |
| CFH Rs800292 F1R2 C/T(r G>A) | Failed, repeat sequencing: failed | CFH isoform precursor NP_000177.2:p.Val62Ile missense variant |  | G=0.68393 |
|  |  |  |  | A=0.31607 |
| CFH Rs1410996 F2R2 C/T(r G>A) | Hetero G/A | Intron variant NA | O | G=0.54453 |
|  |  |  |  | A=0.45547 |
| CFH rs10922109 C>A | Hetero C/A | Intron variant NA | O | C=0.54324 |
|  |  |  |  | A=0.45676 |
| CFH rs1061147 A>C | Hetero A/C | CFH isoform precursor NP_000177.2:p.Ala307= synonymous variant | O | A=0.32473 |
|  |  |  |  | C=0.67527 |
| CFH rs1329428A/G(r C>T) | Hetero T/C | Intron variant NA | O | C=0.56541 |
|  |  |  |  | T=0.43459 |
| C2 Rs9380272 G>A | Homo G | Intron variant | x | ? |
| C2 Rs547154 A/C (r G>T) | Hetero T/G | Intron variant NA, IVS 10; protective | protective | G=0.87956 |
|  |  |  |  | T=0.12044 |
| CFB Rs641153 A/C/T (r G>A / G>T) | hetero G/A | CFB preproprotein NP_001701.2:p.Arg32Gln, NP_001701.2:p.Arg32Leu missense variant | protective | G=0.90424 |
|  |  | R32Q protective |  | A=0.09575, T=0.00000 |
| CFB rs4151667 T>A | Homo T | CFB preproproteinNP_001701.2:p.Leu9His missense variant(L9H); protective | X | T=0.96091 |
|  |  |  |  | A=0.03909 |
| CFB Rs541862 A/G(r T>C) | hetero T/C | Intron variant NA | O | T=0.87890 |
|  |  |  |  | C=0.12110 |
| C3 rs2230199 A/C/G(r G>C / G>T) | Hetero C/G | C3 preproprotein NP_000055.2:p.Arg102Gly, missense variant | O | G=0.84853 |
|  |  |  |  | C=0.15147 |
| CFI rs10033900 T>C | Hetero C/T | Intron variant NA | O | T=0.46107 |
|  |  |  |  | C=0.53893 |

O Pathogenic

X Unknown

**Supplementary Table 2**. Targeting efficiencies of transfected ARPE-19 cells

| **SNP** | **Average Targeting efficiency (%)** |
| --- | --- |
| CFH rs_1410996 | 7.8 |
| CFH rs_380390 | 7.4 |
| CFB rs_641153 (Fw guide 2) | 9.1 |
| CFB rs_641153 (Fw guide 3) | 11.9 |
| CFB rs_641153 (Rv guide 1) | 17.3 |
| CFB rs_641153 (Rv guide 2) | 9.9 |
| CFB rs_541862 | 9.7 |
| C3 rs_147259257 | 15.5 |

**Supplementary Table 3**. sgRNA sequences

| gRNA | Sequence |  |  |
| --- | --- | --- | --- |
| CFH rs1410996-BsmBl-Guide2-F | CACCTGGTATAGCTGAGTGGCATG | | |
| CFH rs1410996-BsmBl-Guide2-R | AAACCATGCCACTCAGCTATACCA | | |
| CFH rs1410996-BsmBl-Guide2-Pathogenic-F | CACCTGGTATAGCTGAGTGACATG | | |
| CFH rs1410996-BsmBl-Guide2-Pathogenic-R | AAACCATGTCACTCAGCTATACCA | | |
| CFH rs1061170-BsmBl-F | CACCAATGGATATAATCAAAATCA | | |
| CFH rs1061170-BsmBl-R | AAACTGATTTTGATTATATCCATT | | |
| CFH rs1061170-BsmBl-Pathogenic-F | CACCAATGGATATAATCAAAATTA | | |
| CFH rs1061170-BsmBl-Pathogenic-R | AAACTAATTTTGATTATATCCATT | | |
| CFB rs5451862-BsmBl-correct-F | CACCTGTGTATCCCTGCCTTTAGCC | | |
| CFB rs5451862-BsmBl-correct-R | AAACGGCTAAAGGCAGGGATACACA | | |
| CFB rs5451862-BsmBl-Pathogenic-F | CACCCGTGTATCCCTGCCTTTAGCC | | |
| CFB rs5451862-BsmBl-Pathogenic-R | AAACGGCTAAAGGCAGGGATACACG | | |
| CFB rs5451862-BsmBl-Guide3-F | CACCTGCCATGTGTATCCCTGCCT | | |
| CFB rs5451862-BsmBl-Guide3-R | AAACAGGCAGGGATACACATGGCA | | |
| CFB rs5451862-BsmBl-Guide3-Pathogenic-F | CACCTGCCACGTGTATCCCTGCCT | | |
| CFB rs5451862-BsmBl-Guide3-Pathogenic-R | AAACAGGCAGGGATACACGTGGCA | | |
| CFB rs641153-BsmBl-3-F | CACCTGGTCTTTGGCCCGGCCCCA | | |
| CFB rs641153-BsmBl-3-R | AAACTGGGGCCGGGCCAAAGACCA | | |
| CFB rs641153-BsmBl-3-Pathogenic-F | CACCTGGTCTTTGGCCCAGCCCCA | | |
| CFB rs641153-BsmBl-3-Pathogenic-R | AAACTGGGGCTGGGCCAAAGACCA | | |
| CFB rs641153-BsmBl-Rev guide1-F | CACCAGAGCAGGATCCCTGGGGCC | | |
| CFB rs641153-BsmBl-Rev guide1-R | AAACGGCCCCAGGGATCCTGCTCT | | |
| CFB rs641153-BsmBl-Rev guide1-Pathogenic-F | CACCAGAGCAGGATCCCTGGGGCT | | |
| CFB rs641153-BsmBl-Rev guide1-Pathogen-R | AAACAGCCCCAGGGATCCTGCTCT | | |

**Supplementary Table 4**. PCR primers sequences

| Primer | Sequence |  |
| --- | --- | --- |
| CFH rs1410996 TIDE Fw | TTGGGAGGCTGAGGAGGAAT | |
| CFH rs1410996 TIDE Rv | AAGCCCTGTATTCTGTCGCT | |
| CFH rs1061170 TIDE F1 | TGATTACACCTGTCTTATG | |
| CFH rs1061170 TIDE R1 | GTCATCTATGTTACTTAG | |
| C3 rs147859257 TIDE F1 | CCTCTGTGTCTCTGCCACTG | |
| C3 rs147859257 TIDE R1 | GCCCCTTCCCTTTCTGAGTC | |
| C3 rs2230199 TIDE F1 | CCTCTGTGTCTCTGCCACTG | |
| C3 rs2230199 TIDE R1 | GCCCCTTCCCTTTCTGAGTC | |
| CFB rs541962 TIDE Fw | TGGTCAAAGGGAAGTCCGTG | |
| CFB rs541862 TIDE Rv | GAAGGATGAGGGTCCAAGCC | |
| CFB rs641153 TIDE Fw | CAAAGCAAGCCAGGACACAC | |
| CFB rs641153 TIDE Rv | GAAACAGCGCATTCCCAGTC | |
